# Neuropathology of Pediatric SARS-CoV-2 Infection in the Forensic Setting: Novel Application of *Ex Vivo* Imaging in Analysis of Brain Microvasculature

**DOI:** 10.3389/fneur.2022.894565

**Published:** 2022-05-24

**Authors:** Michelle N. Stram, Alan C. Seifert, Etty Cortes, Alara Akyatan, Emma Woodoff-Leith, Valeriy Borukhov, Amber Tetlow, Dimath Alyemni, Michael Greenberg, Avneesh Gupta, Amanda Krausert, Lauren Mecca, Sophia Rodriguez, Jay Stahl-Herz, Miguel A. Guzman, Bradley Delman, John F. Crary, Kristen Dams-O'Connor, Rebecca D. Folkerth

**Affiliations:** ^1^Office of Chief Medical Examiner, New York, NY, United States; ^2^Department of Forensic Medicine, New York University Grossman School of Medicine, New York, NY, United States; ^3^Department of Diagnostic, Molecular and Interventional Radiology, Biomedical Engineering and Imaging Institute, Graduate School of Biomedical Sciences, Icahn School of Medicine at Mount Sinai, New York, NY, United States; ^4^Department of Pathology, Molecular and Cell Based Medicine, Mount Sinai Hospital, New York, NY, United States; ^5^Neuropathology Brain Bank & Research Center of Research Excellence (CoRE), Mount Sinai Hospital, New York, NY, United States; ^6^University of New England College of Medicine, Biddeford, ME, United States; ^7^Office of the Medical Investigator, Albuquerque, NM, United States; ^8^Department of Pathology at Saint Louis University School of Medicine, SSM Cardinal Glennon Hospital, St. Louis, MO, United States; ^9^Department of Rehabilitation and Human Performance, Icahn School of Medicine at Mount Sinai, New York, NY, United States; ^10^Department of Neurology, Icahn School of Medicine at Mount Sinai, New York, NY, United States

**Keywords:** COVID-19, MIS-C, encephalitis, microglial nodules, neuroimaging, neuropathology, sudden unexpected infant death, sudden unexpected death in childhood

## Abstract

Two years into the COVID-19 pandemic, there are few published accounts of postmortem SARS-CoV-2 pathology in children. We report 8 such cases (4 infants aged 7–36 weeks, 4 children aged 5–15 years). Four underwent *ex vivo* magnetic resonance neuroimaging, to assist in identification of subtle lesions related to vascular compromise. All infants were found unresponsive (3 in unsafe sleeping conditions); all but 1 had recent rhinitis and/or influenza-like illness (ILI) in the family; 1 had history of sickle cell disease. *Ex vivo* neuroimaging in 1 case revealed white matter (WM) signal hyperintensity and diffuse exaggeration of perivascular spaces, corresponding microscopically to WM mineralization. Neurohistology in the remaining 3 infants variably encompassed WM gliosis and mineralization; brainstem gliosis; perivascular vacuolization; perivascular lymphocytes and brainstem microglia. One had ectopic hippocampal neurons (with pathogenic variant in *DEPDC5*). Among the children, 3 had underlying conditions (e.g., obesity, metabolic disease, autism) and all presented with ILI. Three had laboratory testing suggesting multisystem inflammatory syndrome (MIS-C). Two were hospitalized for critical care including mechanical ventilation and extracorporeal membrane oxygenation (ECMO); one (co-infected with adenovirus) developed right carotid stroke ipsilateral to the ECMO cannula and the other required surgery for an ingested foreign body. Autopsy findings included: acute lung injury in 3 (1 with microthrombi); and one each with diabetic ketoacidosis and cardiac hypertrophy; coronary and cerebral arteritis and aortitis, resembling Kawasaki disease; and neuronal storage and enlarged fatty liver. All 4 children had subtle meningoencephalitis, focally involving the brainstem. On *ex vivo* neuroimaging, 1 had focal pontine susceptibility with corresponding perivascular inflammation/expanded perivascular spaces on histopathology. Results suggest SARS-CoV-2 in infants may present as sudden unexpected infant death, while in older children, signs and symptoms point to severe disease. Underlying conditions may predispose to fatal outcomes. As in adults, the neuropathologic changes may be subtle, with vascular changes such as perivascular vacuolization and gliosis alongside sparse perivascular lymphocytes. Detection of subtle vascular pathology is enhanced by *ex vivo* neuroimaging. Additional analysis of the peripheral/autonomic nervous system and investigation of co-infection in children with COVID-19 is necessary to understand risk for cardiovascular collapse/sudden death.

## Introduction

Infection by the severe acute respiratory syndrome-related novel coronavirus-19 (SARS-CoV-2; COVID-19) emerged at the end of 2019 and quickly evolved into a global pandemic. While initial infection and accompanying neuropathology were recognized primarily in adults [reviewed by (1)], over time it became clear that children were also susceptible, showing, as in adults, a constellation of “influenza-like illness” (ILI) symptoms, such as fever, cough, sore throat, and malaise (2). In many cases, children with COVID-19 suffered a “multisystem inflammatory syndrome in children” (MIS-C), further characterized by elevated serum inflammatory markers, shock, and “echo-bright” coronary vessels, resembling Kawasaki disease (3). Published reports of autopsy pathology of children dying with SARS-CoV-2 infection are limited, and consist of single reports or small series, a subset of which had clinically diagnosed MIS-C (summarized in Table 1). Among those reports, general autopsy findings comprised isolated eosinophilic (hypersensitivity-type) myocarditis in a teenager (4); diffuse alveolar damage and microvascular thrombi in postmortem lung biopsies of 2 infants previously treated with liver transplantation (5), and in infants and school-age children with pre-existing conditions such as adrenal carcinoma, trisomy 18 with congenital heart disease, neonatal cholestasis, sickle-cell trait, disseminated tuberculosis, and out-of-range body-mass index (BMI) (7, 8). Other systemic findings included myocarditis and fibrin thrombi in renal glomeruli (8). One case of a 4-year-old had hospital-acquired superinfection with *P. aeruginosa* and developed pulmonary abscesses (6). Neuropathologic studies are even fewer, but one identified microglial prominence and, rarely, vascular microthrombi (on postmortem needle biopsy only) (8). A singular case study described an 8-year-old who developed unilateral paresis 2 weeks following a febrile illness and underwent brain biopsies and eventual brain-only autopsy (9). Unfortunately, the described and illustrated findings from this case were complicated by limited sampling of frankly ischemic and necrotic brain sites; consequently, the authors' interpretation of “brain vasculitis” is open to question. Of note, essentially all the postmortem neuropathology studies of pediatric COVID-19 published to date were of individuals hospitalized for days to sometimes many weeks, and subject to supervening hypoxic-ischemic or secondary infectious insults.

**Table 1:** Published reports of pediatric SARS-CoV-2 autopsy findings.

**Case**	**Age**	**Prior history**	**Recent history**	**Autopsy findings**	**Neuropathology findings**
Craver et al. (4)	17 y	Previously healthy	2 d history of headache, dizziness, nausea, vomiting; cardiac arrest	Eosinophilic (hypersensitivity) myocarditis	N/A
Imam et al. (5)	10 mo	Cholestatic liver disease	Liver transplant, 8 d PTD, with development of shortness of breath and fever	Early stage DAD with microthrombi in lung (postmortem biopsy only)	N/A
Imam et al. (5)	5 mo	Biliary cirrhosis	Liver transplant, 4 d PTD, with development of progressive respiratory deterioration	Early stage DAD with microthrombi in lung (postmortem biopsy only)	N/A
Menger et al. (6)	4 y	BMI 27.1	Respiratory distress, MIS-C; acute DAD on lung bx at 7 d; progression over 10 d	Necrotizing *P. aeruginosa* pneumonia (superinfection)	N/A
Mulale et al. (7)	3 mo	Coinfection with *M. tuberculosis*	Fever and respiratory distress; progression over 5 d	DAD; microthrombi in lungs and myocardium; disseminated tuberculosis	N/A
Duarte-Neto et al. (8)	15 y	BMI 27.1; metastatic adrenal carcinoma	Hospital-acquired COVID-19 fever and cough, with supervening bacterial pneumonia and systemic thrombosis; progression over 5 d	“Foci” of DAD and bacterial pneumonia with thrombi; hepatic centrilobular necrosis and thrombi with infarcts; ATN; myonecrosis (postmortem needle biopsy only)	Reactive microglia; neuronal ischemia (postmortem needle biopsy only)
Duarte-Neto et al. (8)	7 mo	33 wk preterm; trisomy 18; congenital heart disease; neonatal cholestatic hepatitis; BMI 11.2	Respiratory distress, pneumonia, and multiorgan failure; progression over 12 d	DAD; hepatic cholestasis and centrilobular necrosis; ATN; myonecrosis (postmortem needle biopsy only)	Reactive microglia; neuronal ischemia (postmortem needle biopsy only)
Duarte-Neto et al. (8)	11 y	BMI 22.6; ill relative	Fever, odynophagia, myalgia, abdominal pain, cardiac dysfunction, and MIS-C; progression over 8 d	Foci of pulmonary hemorrhage; microthrombi in lungs and kidney; hepatic centrilobular necrosis; ATN; peri-, myo-, endocarditis; myonecrosis (postmortem needle biopsy only)	Reactive microglia; neuronal ischemia (postmortem needle biopsy only)
Duarte-Neto et al. (8)	8 y	BMI 23.6; asthma; sickle cell trait	Fever, abdominal pain prompting laparotomy, intraoperative shock; MIS-C; progression over 10 d	Foci of DAD; microthrombi in lungs, kidney, and colon; hepatic steatosis; foci of myocarditis and diffuse band necrosis; ATN and hyaline casts; colitis, appendicitis, and peritonitis (surgical specimen); focal myositis (postmortem needle biopsy only)	Reactive microglia; neuronal ischemia; fibrin thrombi (postmortem needle biopsy only)
Duarte-Neto et al. (8)	8 y	BMI 21.24	Fever, odynophagia, headache; progression over 5 d to vomiting and status epilepticus; MIS-C; secondary *S. aureus* pneumonia, death at 28 d	Foci of pulmonary hemorrhage with thrombi; ATN with granular casts; myocardial band necrosis; myonecrosis (postmortem needle biopsy only)	Reactive microglia; neuronal ischemia; Alzheimer type II glia (postmortem needle biopsy only)
Poisson et al. (9)	8 y	Previously healthy	Fever, cough, headache, followed 2 wk later by unilateral weakness; brain biopsies showing necrosis and inflammation at hospital day 4 and weeks later; progression over 93 d	N/A	Right MCA infarct, with necrosis and associated inflammatory changes (as seen in prior biopsies) interpreted as “vasculitis”
Ninan et al. (10)	8 y	Previously healthy	1 d history of fever, lethargy, myalgias, anorexia, seizure-like activity; group A streptococcal pharyngitis and COVID-19; progression to diffuse cerebral edema, pulseless ventricular tachycardia, and brain death over 2 d	Chronic lymphocytic thyroiditis; no arterial or venous thromboses	Diffuse edema with transtentorial herniation, rare acute ischemic neuronal necrosis, and a small number of chronic inflammatory cells in leptomeninges and around few intraparenchymal vessels

*ATN, acute renal tubular necrosis; BMI, body mass index (kg/m^2^; normal range varies with age); d, days; DAD, diffuse alveolar damage; MCA, middle cerebral artery; MIS-C, multisystem inflammatory syndrome in children, as based on clinical appearance and laboratory values; mo, months; N/A, data not available; y, years*.

The goal of this communication is to share the results of *complete* autopsy evaluations, with emphasis on the brain pathology, of predominantly non-hospitalized pediatric cases of SARS-CoV-2 infection encountered in our institution during the initial pandemic period, before the emergence of later coronavirus variants such as omicron. In addition, we applied a novel *ex vivo* magnetic resonance imaging (MRI) protocol in 4 of our cohort, to assist in the identification of subtle lesions as in adult COVID-19 cases previously reported by our group (11).

## Materials and Methods

From March, 2020, through June, 2021, the New York City Office of Chief Medical Examiner (NYC OCME) received 234 pediatric cases (infants and children <18 years of age). Of these, 4 school-age children had known SARS-CoV-2 infection, based on antemortem viral swabs obtained upon their first presentation at health care facilities. Because of our public health mandate for outbreaks of communicable disease, the NYC OCME took jurisdiction over these cases. Four infants came in per our longstanding jurisdictional protocols regarding Sudden Unexpected Infant Death (SUID) and were found to have positive swabs (tested through the New York State Department of Health and Mental Hygiene laboratories, using real-time polymerase-chain reaction techniques for detection of multiple RNA targets in SARS-CoV-2 (as well as influenza A and B) (CDC Multiplex Assay) on postmortem examination. Viral load testing was not indicated in postmortem cases.

All decedents underwent standard autopsy procedures to obtain diagnostic tissue samples, including complete neuropathology. Brain specimens were fixed in 20% formalin for at least 2 weeks before detailed macroscopic examination and photography. Specimens from 4 cases (1 infant, 3 children) were transferred into 10% formalin and fixed for at least 4 weeks prior to undergoing *ex vivo* 3 Tesla magnetic resonance imaging to assist in the identification of subtle or small lesions for diagnostic microscopic evaluation (11).

Immediately prior to imaging, each of the 4 brain specimens was agitated under a vacuum of −1 bar for at least 15 min while immersed in formalin (12) to dislodge air bubbles. Each specimen was then packaged into a custom-built *ex vivo* MRI vessel, which was filled with Fluorinert (FC-770, TMC Industries, Waconia, MN) for magnetic susceptibility matching and elimination of intense MRI signal from formalin (13, 14). Residual formalin, which is less dense than and immiscible with Fluorinert, was removed using a syringe, then the container was sealed and agitated again to dislodge any remaining air and formalin.

Imaging was performed on a 3 Tesla human scanner (Skyra, Siemens Healthineers, Erlangen, Germany) using a 20-channel head/neck coil. The protocol included 3D fluid-attenuated inversion recovery (FLAIR, 0.63 mm isotropic, TR/TI/TE = 4,800/1,650/291 ms, 2.3 h), 3D susceptibility-weighted imaging (0.63 mm isotropic, TR/TE1/TE2 = 29.00/20.00/24.88 ms, 55 min), three 3D multi-echo gradient-echo (ME-GRE) acquisitions at arrayed flip angles (0.39 mm isotropic, TR/TE1/TE2/TE3/TE4 = 28.0/2.95/8.80/14.60/20.40 ms, FA = 45°/25°/10°, 2 h each), and 2D spin-echo echo-planar diffusion imaging (1.8 mm isotropic, TR/TE = 18,000/95 ms, 362 frames as follows: 159 directions at b = 2,000 s/mm^2^ and 22 b = 0 encodings in forward- and reverse-phase encoding directions). ME-GRE images were combined by root-sum-of-squares, and T_1_, T${}_{2}^{*}$, and apparent proton density (S_0_) maps were fitted jointly. Diffusion data were processed in FSL (15). Raw diffusion images were corrected for susceptibility- and eddy current-induced distortions using topup and eddy (16, 17), and fitted to a diffusion tensor model using dtifit.

Each imaging dataset was reviewed by a board-certified neuroradiologist with extensive experience in *ex vivo* MRI technical development. Findings of potential interest for further study were marked and annotated for reference during sectioning. All full field-of-view MR images in figures are displayed in radiological orientation convention unless otherwise indicated by L/R labels superimposed on the images.

All brains were examined by study neuropathologists, and sectioned and sampled for microscopy. The imaged brains were subsequently embedded in 3% agar and sectioned using a rotary blade (0.4 cm) at the Mount Sinai Neuropathology Brain Bank & Research CoRE facility, while the others were sectioned at the NYC OCME using standardized protocols. Histologic processing and staining were performed using standard diagnostic methods. Hematoxylin and eosin (H&E) or Luxol fast blue (LFB)/H&E stains were employed in all cases. Immunostains for inflammatory cells {CD68 [Cell Marque (catalog # 168M-98), pre-diluted]; IBA1 [Invitrogen (catalog # PA5-27436), dilution 1:1,000]; CD3 [Leica (catalog # PA0122), pre-diluted]; CD20 [Leica (catalog # PA0359), pre-diluted]} and SARS-CoV-2 spike proteins [PreSci (catalog # 3525), dilution 1:12,000)] on selected brain tissue blocks in Cases 6 and 8.

Because of the exploratory nature of this work, and the relatively limited numbers of cases, no statistical analyses were applied.

## Results

The clinical features and findings at autopsy are summarized in Table 2. To protect the privacy of the children and their families, we provide limited information regarding their age, sex, race, and ethnicity. The cases distributed into 2 groups: infants (<1 year of age) presenting with sudden unexpected death (SUID) (*n* = 4), and children of school-age (>4 years old) (*n* = 4), 3 of whom had varying past medical histories, including obesity, autism, and systemic enzyme deficiency. The male:female ratio was equal. Four subjects were Black/African American; 2 were of Hispanic ethnicity, 1 was of Asian/Pacific extraction, while 1 had no race/ethnicity specified.

**Table 2:** Clinical characteristics, *in vivo* CT and *ex vivo* MR neuroimaging, and autopsy findings in this cohort.

**Case**	**Prior history**	**Recent history**	**Autopsy findings**	**Neuroimaging**	**NP findings**
1	Term C-section delivery for maternal diabetes	Recent rhinitis; SUID in unsafe sleep environment with positional asphyxia	Postmortem swab also + for rhino/enterovirus nucleic acid	*Ex vivo* MRI: broad areas of WM signal hyperintensity and diffuse exaggeration of perivascular spaces (Figure 1)	Microscopic multifocal WM mineralization (Figure 1)
2	ILI in parents and other family in past weeks	SUID in unsafe sleep environment	Likely pathogenic variant in *DEPDC5* (associated with familial epilepsy)	N/A	Microscopic WM mineralization and gliosis, ectopic neurons in hippocampus
3	Sickle cell disease (homozygous HgbS); ILI in parents in past weeks	SUID	Interstitial pneumonitis with microthrombi	N/A	Microscopic WM and brainstem gliosis, acute neuronal ischemia
4	Treated for presumed congenital syphilis (mother incompletely treated)	SUID in unsafe sleep environment	No systemic cause of death, besides NP findings	N/A	Microscopic multifocal perivascular vacuolization and gliosis and sparse brainstem inflammation (Figure 1), perivascular lymphocytes and mineralization in basal ganglia
5	Speech delay	Fever, cough, nasal congestion, abdominal pain, diarrhea; MIS-C; co-infection with adenovirus; treatment with vent and ECMO in hospital for 11 d	DAD; hypersensitivity myocarditis; coronary arteritis and aortitis (Figure 3)	*In vivo* CT: R MCA and ACA infarct and diffuse L subarachnoid hemorrhage (Figure 2)	R MCA and ACA infarct (ipsilateral to ECMO); microscopic L MCA arteritis, perivascular lymphocytes in non-infarcted parenchyma, necrosis and inflammation in autonomic ganglia, peripheral nerve, skeletal muscle (Figures 2, 3)
6	BMI 29; asthma	Fever; MIS-C; collapse and death in ED after few hours survival	DAD; cardiac hypertrophy	*Ex vivo* MRI: linear region of susceptibility-induced T${}_{2}^{*}$ shortening in the medulla (Figure 4); focal vascular cluster in medial R occipital lobe suggesting DVA (Figure 5)	Microscopic early non-occlusive thrombus in leptomeningeal venule, sparse microglial nodules in medulla (Figure 4)
7	Metabolic (enzyme deficiency) disorder; pica	Fever; MIS-C; vomiting, surgery for ingested foreign body; treatment with vent and ECMO in hospital for 4 d	DAD; peritonitis; conjunctivitis	*Ex vivo* MRI: focal pontine susceptibility (Figure 6), vague parietal periventricular WM signal hyperintensity in non-specific distribution, and several DVA	Microscopic gliosis and neuronal loss in thalamus, perivascular lymphocytes, edema, microglial nodules in pons (Figure 6)
8	BMI 45.1; autism	Fever, ataxia; collapse and death in ED shortly after arrival	Diabetic ketoacidosis; possible DAD (resuscitation changes); cardiac hypertrophy	*Ex vivo* MRI: focal periventricular region of susceptibility-induced T${}_{2}^{*}$ shortening (Figure 7)	Microscopic periventricular and perivascular mineralization in WM; sparse lymphocytes in brain and meninges, microglial aggregates (Figure 7)

*Cases 1–4 were infants aged 7 weeks to 4 months; Cases 5–8 were school-age children aged 5–15 years. ACA, anterior cerebral artery; BMI, body mass index (kg/m^2^; normal range varies with age); CT, computed tomography; d, days; DAD, diffuse alveolar damage; DVA, developmental venous anomaly; ECMO, extracorporeal membrane oxygenation; ED, Emergency Department; HgbS, hemoglobin S gene allele; ILI, influenza-like illness; L, left-sided; MCA, middle cerebral artery; MISC-C, multisystem inflammatory syndrome in children, as based on clinical appearance and laboratory values; NP, neuropathology; R, right-sided; SUID, sudden unexpected infant death; vent, mechanical ventilation; WM, white matter*.

### Infants (Cases 1–4)

**Figure 1:**
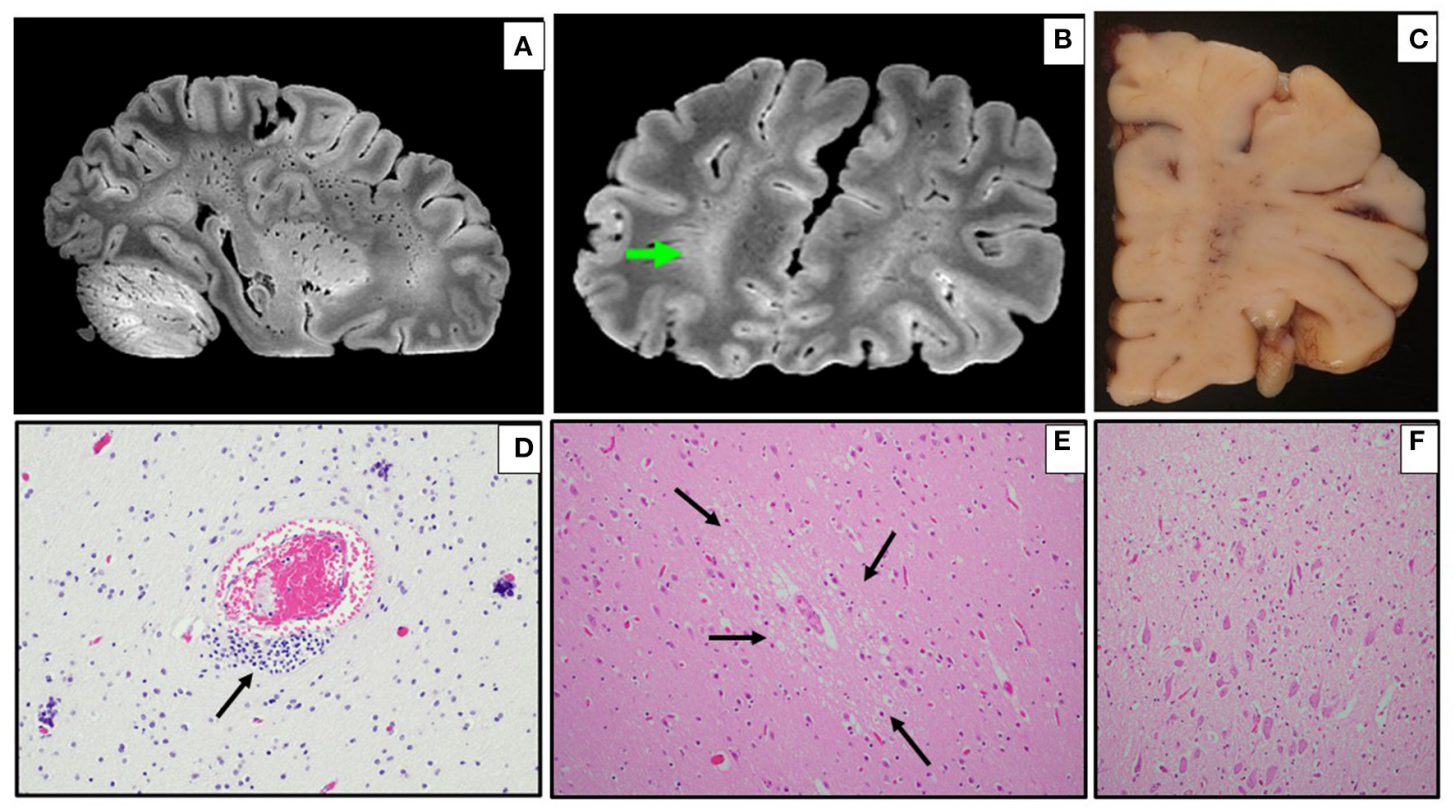
*Ex vivo* MRI and neuropathology in infants (Cases 1–4). **(A,B)** Sagittal **(A)** and coronal **(B)** multi-echo gradient-echo MRI with diffuse exaggeration of perivascular spaces, and broad areas of white matter signal hyperintensity [green arrow in right frontal lobe, per radiological orientation convention (patient's right on the left of the screen) **(B)**] (Case 1). **(C)** Coronal section of right frontal lobe showing prominent white matter vasculature (Case 1). **(D)** Multifocal perivascular mineralization in frontal white matter, as well as residual immature cells (arrow) (H&E, 200x) (Case 1). **(E)** Multifocal perivascular vacuolization (arrows) and gliosis in gray matter (H&E, 200x) (Case 4). **(F)** Sparse parenchymal inflammation in brainstem (H&E, 100x) (Case 4).

**Figure 2:**
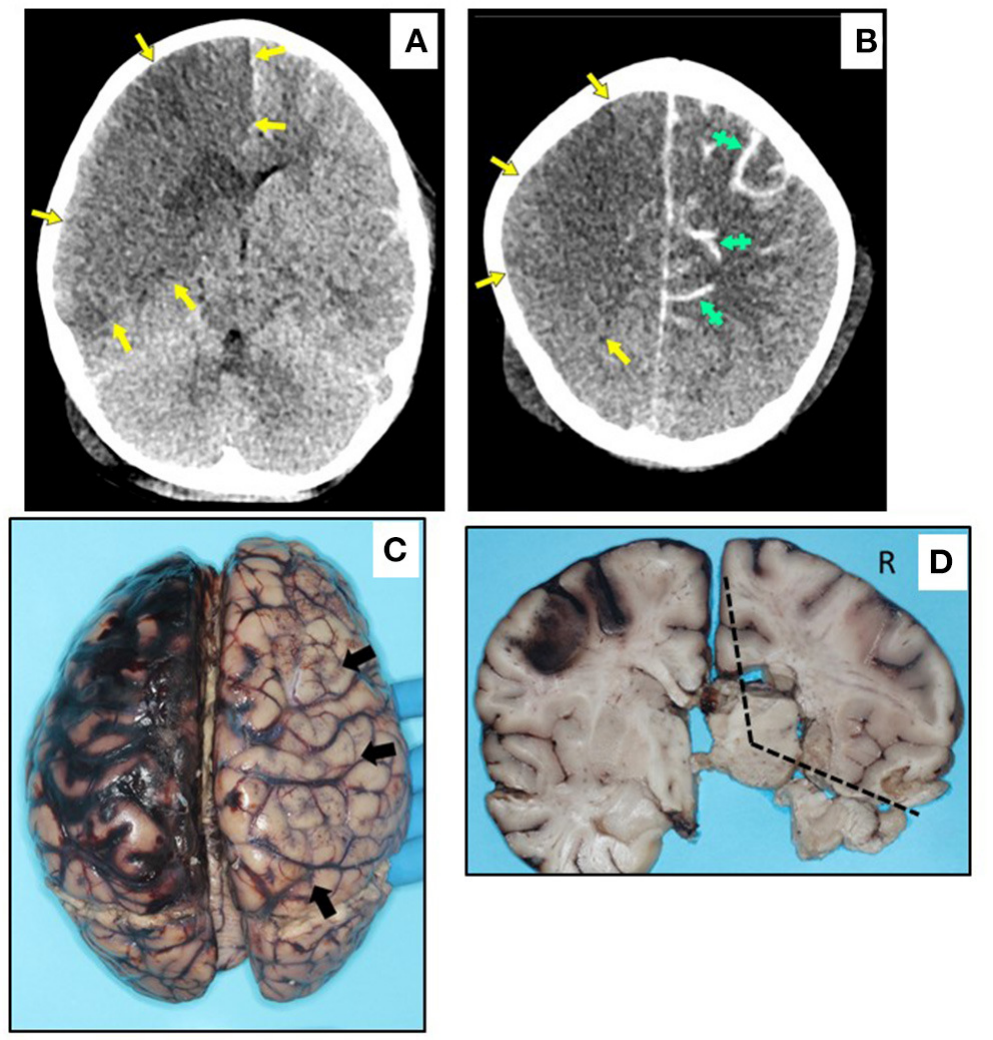
*In vivo* CT imaging and macroscopic neuropathology in Case 5. **(A,B)** Axial non-contrast CT images, displayed in radiological orientation convention. **(A)** Broad area of hypodensity throughout entire right MCA and ACA distributions (yellow arrows) at level of mid-basal ganglia, reflecting cytotoxic edema in a broad area of infarction; areas of even lower density in caudate head and anterior lenticular nucleus, suggesting older or more established infarcts; minimal midline shift despite infarct volume. **(B)** Right MCA and ACA infarct at level of upper centrum semiovale, extending more superiorly to the vertex (yellow arrows); additional parenchymal hypodensity on left side, with increased sulcal density (green hatched arrows) reflecting diffuse subarachnoid hemorrhage. **(C)** Dorsal view of brain, with acute right carotid territory infarct (arrows), as well as subarachnoid hemorrhage over left hemisphere. **(D)** Coronal section at level of basal ganglia, showing infarct (R = right, dotted lines), and hypoperfusion of left watershed zone with reperfusion hemorrhage.

Recent histories of ILI were obtained in family members (SARS-CoV-2 testing status unknown) in Cases 1 and 2, and Case 1 had recent rhinitis. Case 2 had sickle-cell disease, and Case 4 had been treated at birth for possible congenital syphilis (mother incompletely treated). The general autopsy findings were limited. The infant with rhinitis (Case 1) had, in addition to SARS-CoV-2, postmortem viral swab detection of rhino/enterovirus nuclei acid. Case 2 had a likely pathogenic variant in *DEPDC5* (a gene associated with familial epilepsy) on molecular genetic testing, although the role of this in the death of the infant was uncertain [of note, we did find ectopic neurons in the hippocampus, as has been seen in sudden unexpected death in toddlers with personal or family histories of febrile seizures (18). Case 3 (the infant with sickle-cell disease) had interstitial pneumonitis with fibrin thrombi. Case 4 had no alternative anatomic cause of death on general organ evaluation. Macroscopically, Case 4 had foreshortening of the frontal lobes, and small superior temporal gyri; the remaining infant brains were macroscopically normal. *Ex vivo* MRI in Case 1 showed broad areas of white matter signal hyperintensity and diffuse exaggeration of perivascular spaces atypical for an infant on ME-GRE (Figure 1). Microscopic findings comprised multifocal white matter mineralization in Cases 1 (Figure 1) and 2, with accompanying gliosis in the latter (not shown). Case 3 had microscopic evidence of white matter and brainstem gliosis, as well as acute neuronal ischemia (not shown). Of note, Case 4 had multifocal perivascular vacuolization and reactive changes, suggesting vasogenic edema and/or vascular “leakiness”; sparse perivascular lymphocytes were also noted in the basal ganglia, with prominent groups of microglia and mononuclear cells noted in the brainstem (Figure 1).

### Children (Cases 5–8)

**Figure 3:**
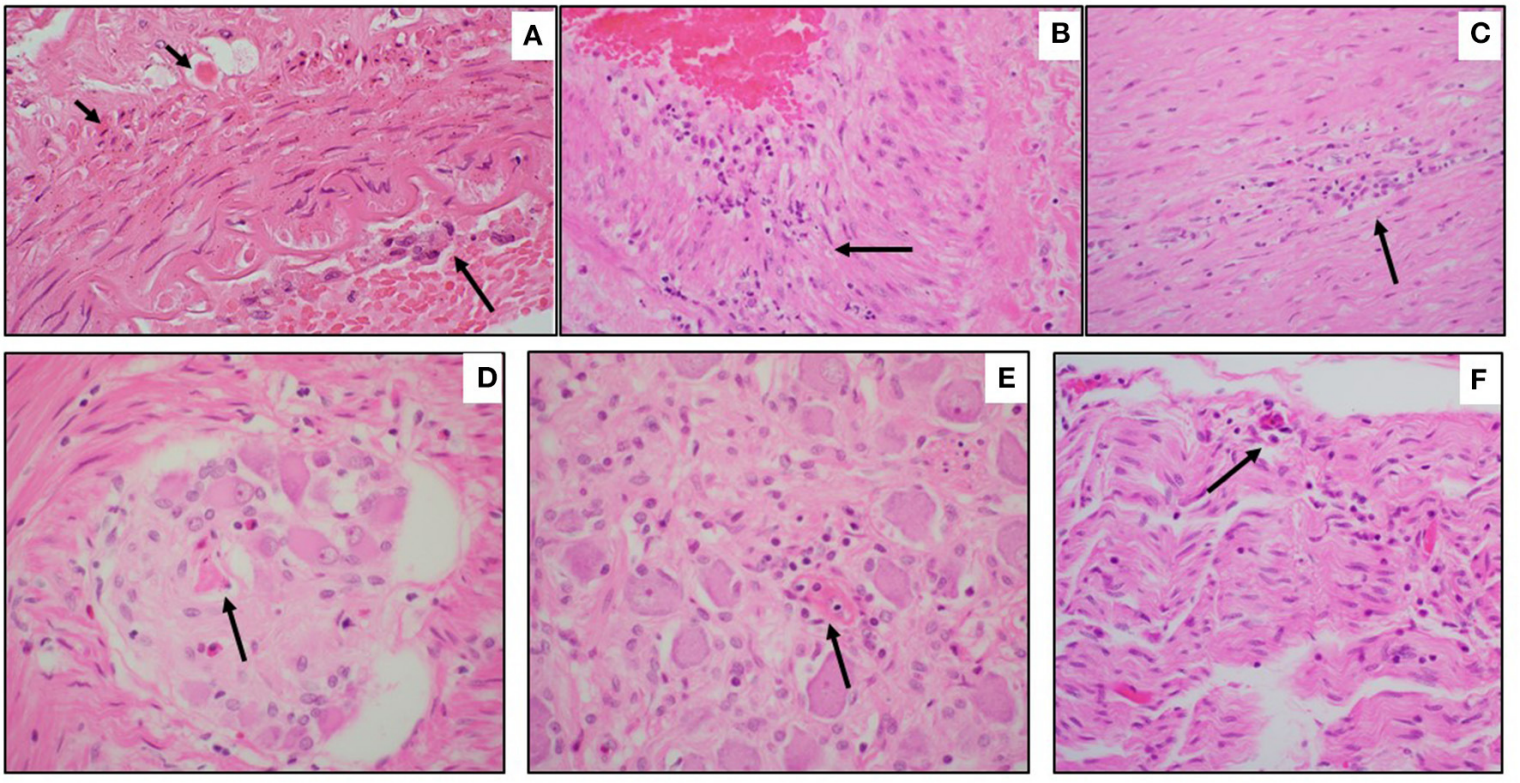
Microscopic neuropathology in Case 5. **(A)** Left MCA with arteritis, characterized by endotheliitis (long arrow) and individually necrotic vascular smooth muscle cells (short arrows) (H&E, 200x). **(B)** Coronary arteritis, with full-thickness mural inflammation (arrow) (H&E, 200x). **(C)** Aortitis, with mural inflammation (arrow) (H&E, 100x). **(D)** Myenteric ganglionitis, with necrotic neuron (arrow) (H&E, 400x); **(E)** Sympathetic ganglionitis, with rare fibrinoid vessel (arrow) (H&E, 200x); **(F)** Celiac nerve with inflammation and endothelial prominence (arrow) (H&E, 100x).

Pre-existing conditions of obesity (Case 6 and 8), asthma (Case 6), metabolic disorder (Case 7), and autism (Case 7 and 8) were noted. Fever was the common presenting symptom across these cases. Three children (Cases 5, 6, and 7) had symptoms and laboratory features of MIS-C, and Cases 6 and 8 collapsed and died shortly after arrival at the hospital Emergency Department. Cases 5 and 7 had hospital courses of 11 and 4 days, respectively, both marked by the need for mechanical ventilation and extracorporeal membrane oxygenation (ECMO) for refractory hypoxemia. Case 5 developed hypotension with cardiogenic shock secondary to inflammatory cytokine storm. This child developed a right carotid artery territory infarct on the same side as the ECMO cannula (Figure 2), and was also found to have co-infection with adenovirus. Case 7 required gastrointestinal surgery for ingestion of a foreign object, with associated abdominal complications. At autopsy, there was acute lung injury in Cases 5, 6, and 7; in Case 8, some microscopic lung changes could not be distinguished from resuscitation. Case 8 was found at autopsy to have diabetic ketoacidosis (without antemortem diagnosis of diabetes mellitus), based on postmortem vitreous glucose of 479 mg/dl, as well as serum acetone levels of 0.010 g% and elevated beta-hydroxybutyrate. The 2 obese children, including the one with diabetic ketoacidosis, had cardiac hypertrophy. Of note, Case 5 had coronary arteritis and aortitis, reminiscent of changes seen in Kawasaki disease (Figure 3).

On brain autopsy, Cases 6, 7, and 8 had cloudiness of the leptomeninges macroscopically (not shown). In Case 5, the right carotid artery infarct and left subarachnoid hemorrhage noted on antemortem CT were confirmed macroscopically; the left subarachnoid hemorrhage originated from reperfusion hemorrhage in the anterior-middle cerebral artery border (“watershed”) zone (Figure 2). Notably, the left middle cerebral artery (contralateral to the infarct) showed Kawasaki-like changes, as well as perivascular lymphocytic inflammation in non-infarcted brain (Figure 3). A remarkable finding in Case 5 was focal necrosis of neurons in several autonomic ganglia, with focal fibrinoid microvascular change, and sparse inflammation in peripheral nerves (Figure 3) and skeletal muscle (not shown).

**Figure 4:**
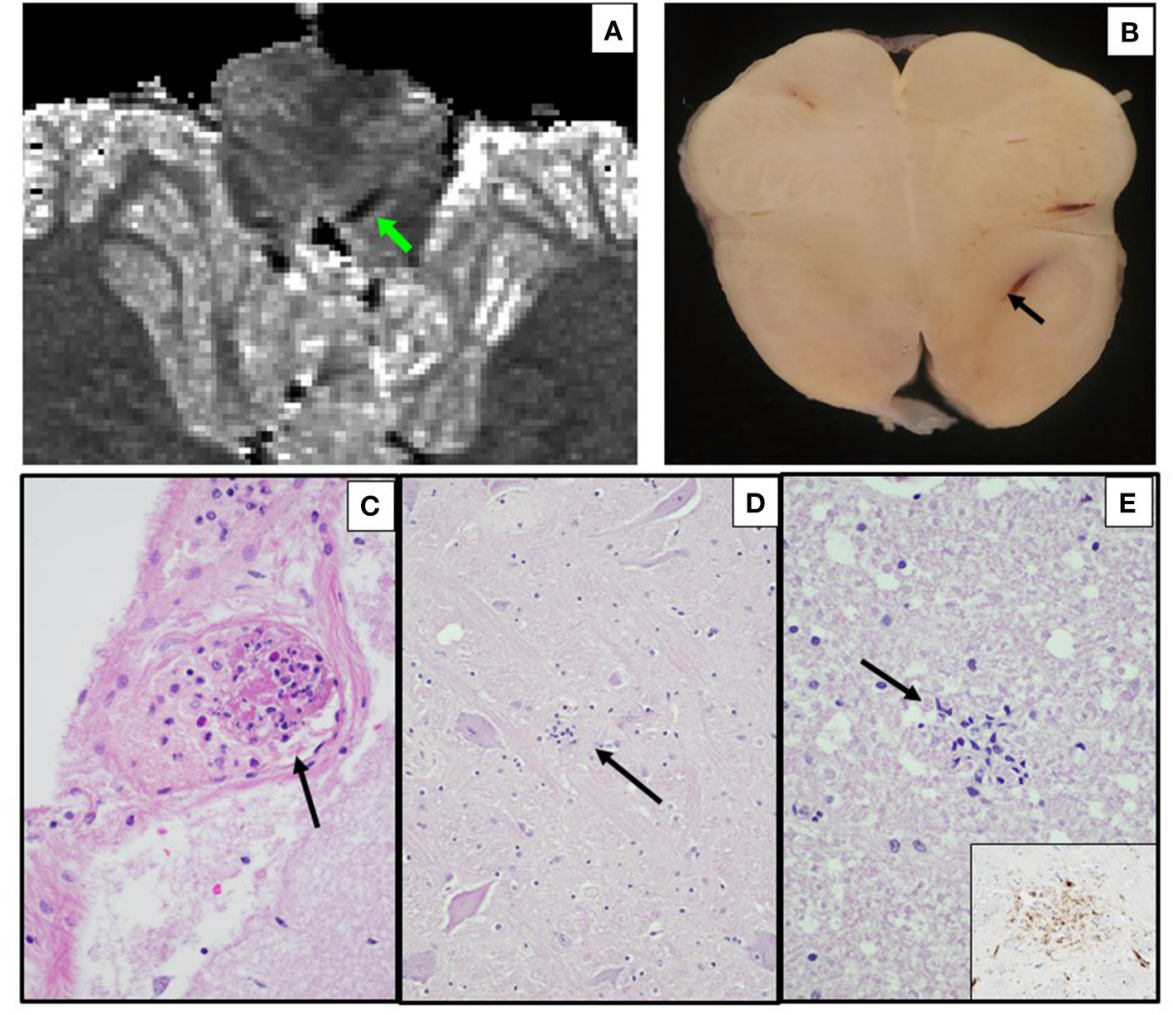
*Ex vivo* MRI and autopsy neuropathology in Case 6. **(A)** *Ex-vivo* axial T${}_{2}^{*}$ parametric map derived from multi-echo gradient-echo MRI, showing a linear region of susceptibility-induced T${}_{2}^{*}$ shortening in the medulla. **(B)** Corresponding axial section of the medulla, with same prominent parenchymal vein (arrow). **(C)** Leptomeningeal venule containing fibrin and inflammatory cells (early non-occlusive thrombus) (arrow) (H&E, 400x). **(D)** Medulla with microglial nodule (arrow) (H&E, 200x). **(E)** Cervical spinal lateral funiculus microglial nodule (H&E, 400x; inset, CD68 immunostain, 400x).

**Figure 5:**
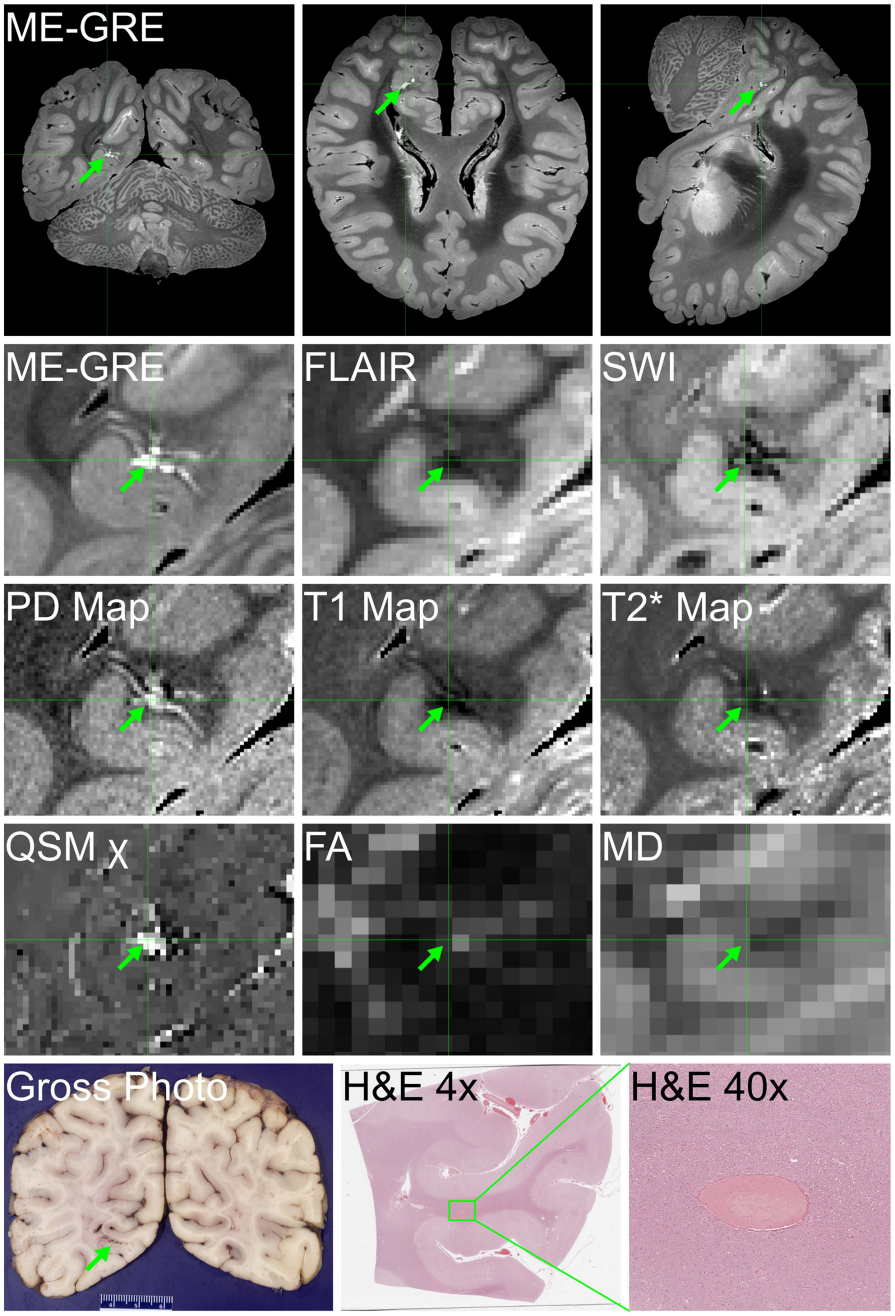
*Ex vivo* MRI and autopsy neuropathology in Case 6. *Ex vivo* MRI findings of hyperintensity on multi-echo gradient-echo (ME-GRE), hypointensity on FLAIR and SWI, T_1_ and T${}_{2}^{*}$ shortening, and high proton density (PD) and susceptibility (χ) in the right occipital lobe, consistent with a developmental venous anomaly, histologically confirmed, attesting to our ability to distinguish COVID-19-related findings from other pathologic, incidental findings in *ex vivo* MRI.

**Figure 6:**
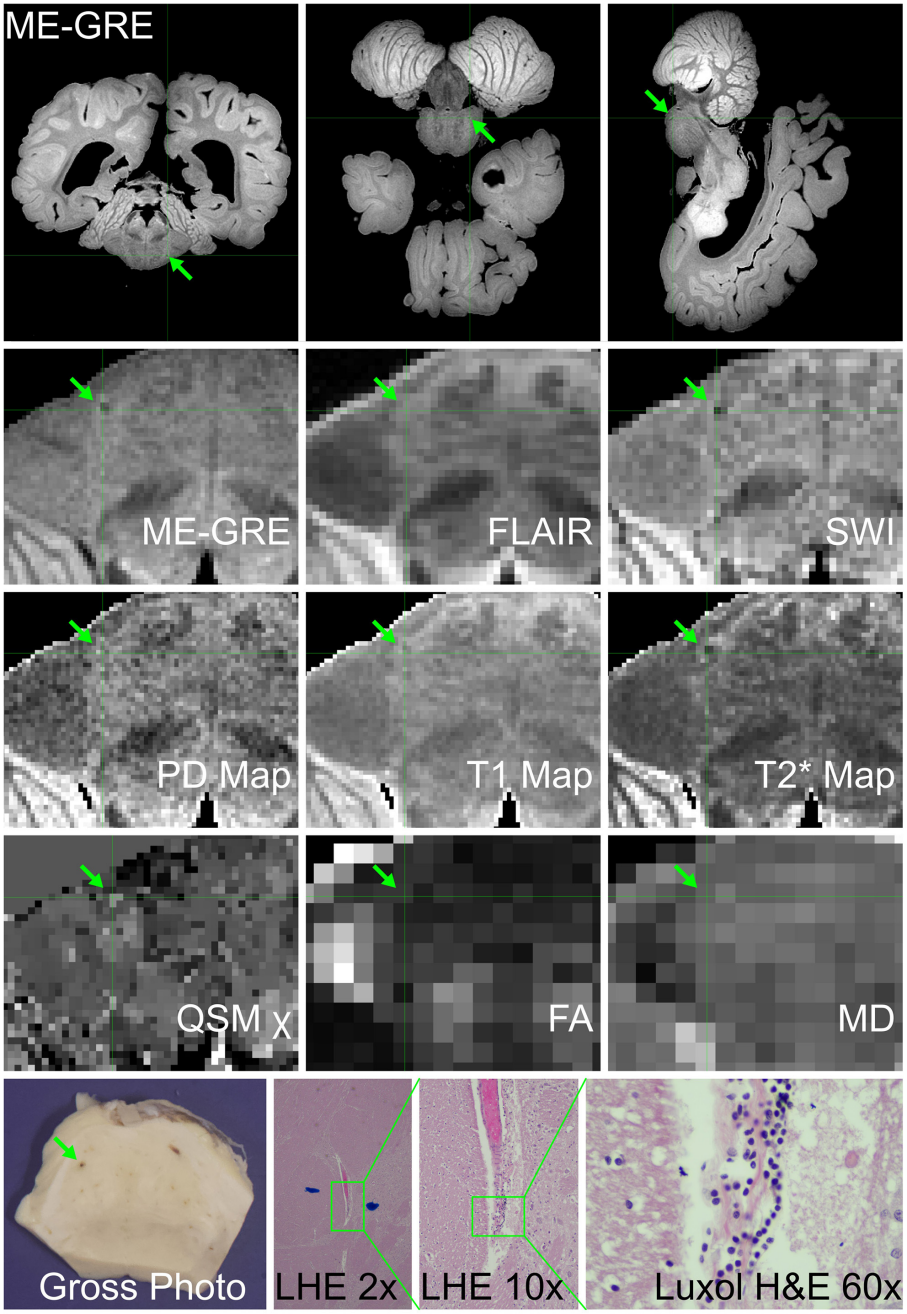
*Ex vivo* MRI and autopsy neuropathology in Case 7. **(Top)** Three-plane multi-echo gradient-echo MR images, displayed in radiological orientation convention, showing the location of the imaging-identified finding in the pons. **(Middle)** The appearance of the finding of interest on multiple image contrasts and parametric maps, displayed in radiological orientation convention maps. **(Bottom)** Gross photograph of pons with corresponding blood vessel; Luxol fast blue H&E (LHE) histology of same vessel, which at higher magnification has perivascular rarefaction, proteinaceous material and lymphocytes (ocular magnificaitons 2 ×, 10 ×, and 60 ×, respectively).

**Figure 7:**
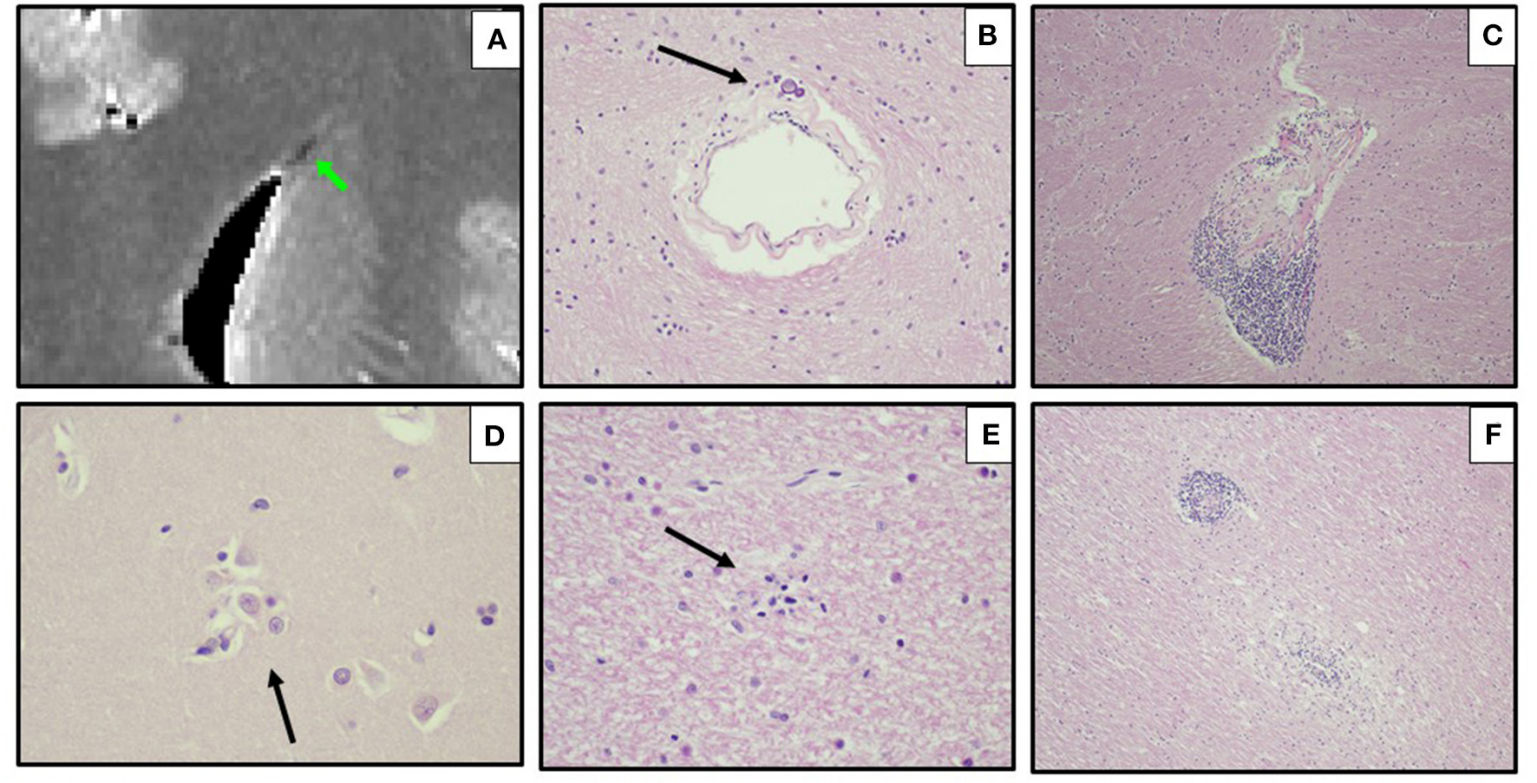
*Ex vivo* MRI and autopsy neuropathology in Case 8. **(A)** *Ex-vivo* coronal T${}_{2}^{*}$ parametric map derived from multi-echo gradient-echo MRI at the cingulum, displayed in radiological orientation convention, showing a focal periventricular region of susceptibility-induced T${}_{2}^{*}$ shortening (green arrow). **(B)** Thickened periventricular vessel with mineralization, atypical for child of this age (H&E, 200x). **(C)** Internal capsule with perivascular lymphocytes (arrow) (H&E, 100x). **(D)** Putamen (600x), and **(E)** subcortical white matter (400x) with microglial nodules (arrows) (H&E). **(F)** Pons, caudal, with perivascular and parenchymal mononuclear inflammation (H&E 100x).

*Ex vivo* MRI of Case 6 showed a linear region of susceptibility-induced T${}_{2}^{*}$ shortening and hypointensity on ME-GRE and SWI in the medulla (Figure 4); this finding was not visible on FLAIR. A focal hyperintensity on ME-GRE and hypointensity on FLAIR and SWI was also found in the occipital lobe, consistent with a developmental venous anomaly, which was confirmed on neuropathology (Figure 5). Case 7 was notable for a focal short-T${}_{2}^{*}$ hypointensity on ME-GRE and FLAIR images in the pons (Figure 6), as well as regions of white matter hyperintensity on FLAIR and T_1_, T${}_{2}^{*}$, and proton density parametric maps. These changes corresponded to perivascular inflammation and proteinaceous edema fluid neuropathologically (Figure 6). *Ex vivo* MRI in Case 8 showed focal periventricular white matter finding of increased proton density, shortened T${}_{2}^{*}$, hypointensity on ME-GRE and SWI, and hyperintensity on FLAIR (Figure 7), In the periventricular white matter Scattered focally prominent perivascular spaces were also present.

On neurohistology, parenchymal microglial aggregates (confirmed on CD68-immunostains in Cases 6–8; Figure 4) indicated subtle encephalitis (Figures 3, 4, 6). Immunostaining for CD3 (T-cells) and CD20 [B-cells] confirmed the sparse predominantly T-cell mononuclear infiltrates, and no positivity for COVID-19 spike protein (Cases 6–8) (not shown).

## Discussion

Herein we share our findings, including complete autopsy, in 4 infants and 4 school-age children dying with confirmed SARS-CoV-2 infection during the initial 17 months of the pandemic in a large urban forensic office. We also provide results of a novel *ex vivo* MRI protocol applied to 4 of our cases and demonstrate its utility in identifying subtle brain abnormalities, particularly involving the microvasculature. These subtle findings were most often identified on ME-GRE, SWI, and T${}_{2}^{*}$ relaxometry, and somewhat less often on FLAIR, highlighting the importance of multi-contrast *ex vivo* MRI datasets for this purpose.

Clinically, the infants were referred to our office as sudden unexpected deaths in infancy (SUID), per statute, and underwent our full examination protocol, including nasopharyngeal swabbing for a number of respiratory viruses, to which SARS-CoV-2 was added in March, 2020. The autopsy changes were subtle among this group, and required synthesis with the individual clinical circumstances, as is routine for sudden unexpected infant demises. Features such as interstitial pneumonitis, white matter and brainstem gliosis, and mineralization (as seen in some of our infants) are well-recognized in SUID as abnormal while still being insufficient causes of death on their own, further reinforcing the need for microbiologic testing as part of a complete infant autopsy (19). Our case with known sickle-cell disease and pulmonary fibrin thrombi recalls 4 reported infant cases of COVID-19 with pulmonary microthrombi. However, our 4 infants did not have acute lung injury (diffuse alveolar damage) as described in iatrogenically immunocompromised 5- and 10-month-old infants after liver transplantation (5), in a 3-month-old with disseminated tuberculosis (7), and in a 7-month-old with multiple congenital anomalies (8). Note that only 1 of those reported infants had a neuropathologic examination (needle biopsy) showing microglia and neuronal ischemia (8).

The occurrence of unsafe sleeping conditions among our infants unfortunately is not at all unusual in our urban setting, and in 1 case was actually of sufficient degree, based upon death scene investigation, to result in a determination of asphyxia as the cause of death. Thus, we must consider that the SARS-CoV-2 positivity in our infant cohort is coincidental rather than a cause of death *per se*, although the microscopic findings of subtle inflammation are not usual in the brain in sudden infant death syndrome. Of note, the combination of unrecognized infection and suboptimal sleep environment has long been proposed to act synergistically to cause fatal compromise in sudden unexpected infant deaths (19). We urge awareness of this possibility among our forensic colleagues.

The autopsy changes in our 4 older children, who were clearly clinically ill, resemble somewhat those reported by other authors in single cases or small case series, specifically acute lung injury, focal microthrombi in lungs and occasionally other organs, and myocarditis (4, 8). Of note, some of the reported children had pre-existing medical conditions including out-of-range BMI (most frequently obesity as seen in 2 of our cohort, but undernutrition in 1 reported case). Superimposed bacterial pneumonia in a 4-year-old (6) and in a 15-year-old (8) likely reflect secondary complications of hospitalization, not seen in our 2 previously hospitalized patients. Likewise, acute renal tubular necrosis, myocardial necrosis, and skeletal muscle necrosis as seen in 5 hospitalized cases (8) were not features of our cohort.

The published neuropathologic findings among school-age children, when available, were relatively non-specific, although consistent with systemic infection and/or hypoxia-ischemia which often engender reactive microglia (8). While we identified that as well, we further demonstrated parenchymal perivascular T-cell lymphocytic infiltrates and microglial aggregates, though sparse overall. The 8-year-old described by Poisson et al. (9) raised very interesting points regarding the presumption of underlying SARS-CoV-2-related “vasculitis” as the cause of a middle cerebral artery infarct. Unfortunately, they of necessity had only biopsies of the infarcted regions during life, and on brain autopsy after 93 days described inflammation and vascular changes only within necrotic and reactive areas (which would be expected in an organizing infarct). Thus, their report stands in contrast to our case in which we clearly confirm a known recent infarct (ipsilateral to the ECMO cannula, and therefore indeterminate as to cause) but with *contralateral* cerebral arteritis (i.e., on the non-necrotic side), as well as coronary arteritis and aortitis. Our cases further add emphasis to the importance of recognition of the clinical signs, symptoms, and serum inflammatory markers of MIS-C in COVID-19 among school-age children.

In conclusion, we have shown that pediatric COVID-19 has subtle but significant findings on complete autopsy, and that, as in adults, underlying medical conditions, particularly obesity, may predispose to fatal outcomes. While this case series does not permit quantification of COVID-19 mortality risk or investigation of postmortem features across racial or ethnic groups, it is noted that all but one case is from a racial or ethnic minority group. Co-infection with a second virus should be sought, and laboratory evaluation for MIS-C conducted proactively. Distinctive findings include Kawasaki-like changes of arteritis and aortitis, and peripheral and autonomic nervous system inflammation. The latter begs the question of a potential substrate for cardiovascular collapse/sudden death in the population coming to forensic autopsy. Of particular note, our employment of the novel technique of multi-contrast *ex vivo* MRI of brain specimens in 1 infant and 3 childhood cases highlighted subtle but likely physiologically significant microvascular changes confirmed on neurohistology, which were strikingly similar to those reported previously in adults with COVID-19, many of whom died suddenly, by our group (11).

## Data Availability Statement

The original contributions presented in the study are included in the article/supplementary material, further inquiries can be directed to the corresponding author/s.

## Ethics Statement

Ethical review and approval was not required for the study on human participants in accordance with the local legislation and institutional requirements. Written informed consent from the participants' legal guardian/next of kin was not required to participate in this study in accordance with the national legislation and the institutional requirements.

## Author Contributions

MS, AS, EC, BD, JC, KD-O'C, and RF: conception, design, execution of study, and drafting of manuscript. MS, EC, DA, MG, AG, AK, LM, SR, JS-H, MAG, JC, and RF: pathological analyses. AS and BD: neuroimaging analyses. AA, EW-L, VB, and AT: technical assistance. All authors contributed to the article and approved the submitted version.

## Funding

This work was supported by the National Institutes of Health [RF1MH128969, R01 AG054008, R01 NS095252, R01 NS086736, and R01 AG062348 to JC], the Rainwater Charitable Trust (Tau Consortium), National Institute of Neurological Disorders and Stroke (NINDS)/National Institutes of Health (NIH) 1RF1NS115268 (KD-O'C), and NINDS K01NS105160. This work was performed using the Multi-Band Accelerated EPI Pulse Sequence package provided by the University of Minnesota Center for Magnetic Resonance Research (AS).

## Conflict of Interest

The authors declare that the research was conducted in the absence of any commercial or financial relationships that could be construed as a potential conflict of interest.

## Publisher's Note

All claims expressed in this article are solely those of the authors and do not necessarily represent those of their affiliated organizations, or those of the publisher, the editors and the reviewers. Any product that may be evaluated in this article, or claim that may be made by its manufacturer, is not guaranteed or endorsed by the publisher.
